# Pitfalls and Risks of “New Eating Disorders”: Let the Expert Speak!

**DOI:** 10.3390/nu15061307

**Published:** 2023-03-07

**Authors:** Alessia Salatto, Maria Pia Riccio, Raffaele Garotti, Carmela Bravaccio, Maria Immacolata Spagnuolo

**Affiliations:** 1 Department of Pediatrics, University Federico II of Naples, Via Sergio Pansini 5, 80131 Naples, Italy; 2 U.O.S.D. of Child Neuropsychiatry, Department of Translational Medical Sciences, 80131 Naples, Italy

**Keywords:** eating disorder, atypical anorexia, ARFID, children, red flags, diagnostic clues

## Abstract

Since the post-pandemic period, there has been an increase in the incidence of eating disorders (EADs) and a lowering of the age of onset. In addition to the ‘classic’ forms, there has also been an increase in new forms of EADs. This article proposes a brief review of the literature concerning mainly two of these new disorders: atypical anorexia and avoidant/restrictive food intake disorder. In addition, a brief overview is proposed of the most frequently raised questions that clinicians may face when dealing with EADs. The answers are provided by doctors from the Federico II University of Naples, who additionally offer the most common red flags on the topic derived from long clinical experience. This article is proposed to be a brief operational guide for all clinicians working in the pediatric area in order to provide diagnostic clues and useful elements to refer patients to specialists for a correct and multidisciplinary treatment.

## 1. Introduction

Eating disorders (EADs) represent a large and complex chapter of psychopathology as they present a great transversality in terms of nosography and age of presentation. The severity of EADs depends on the high risk of chronicity and recurrence, as well as the consequences of the resulting malnutrition. Mortality rates, both direct and indirect (i.e., due to other comorbidities, including psychiatric, e.g., suicidality) are high [1]. With the fifth edition of the Statistical and Diagnostic Manual of Mental Disorders (DSM-5, American Psychiatric Association, APA 2013) [2], the concept of an eating disorder is broadened and becomes more complex and articulated: not just ‘behavioral’ disorders, but more correctly refers to ‘Nutrition and Eating Disorders’, referring to all those conditions characterized by a ‘*persistent eating disorder or eating-related behaviors that result in impaired food consumption or absorption and significantly impair physical health or psychosocial functioning*’. The DSM-5, therefore, makes more explicit the interconnection between the psychiatric aspect and the physical consequences of these complex conditions and expands the category. In particular, the inclusion of avoidant/restrictive food intake disorder (ARFID), an eating disorder predominantly found in children, emphasizes a condition that, although not underpinned by a thought associated with weight or body shape, involves low weight, a state of malnutrition and dysfunctioning in psychosocial terms, is also to be considered a psychiatric disorder. Moreover, since the post-pandemic period, an increase in the incidence of EADs occurred and a decrease in age of onset; in particular, ‘partial’ or not otherwise specified (NOS) or atypical forms have increased; these conditions differ from other EADs, also because they tend to occur in both sexes and can occur early in childhood [3]. Early identification and treatment of EADs are certainly associated with a better outcome [4]. Indeed, in order to provide adequate care for persons suffering from psychiatric disorders, especially in childhood, an accurate diagnosis and adequate assessment of therapeutic need is necessary for appropriate and evidence-based treatment. However, psychiatric disorders, including EADs, are often not promptly or appropriately recognized, thus remain untreated. The diagnosis of specific pictures of EADs is complex to make and when it is appropriate, access to treatment is often extremely difficult given the paucity of available services where evidence-based treatment is available [5]. A large number of people suffering from EADs fail to access adequate treatment services and the majority of people suffering from an EAD remain undiagnosed or untreated, resulting in chronic states and permanent disabilities of both a psychiatric and social, as well as a medical-organic nature [6,7]. Therefore, developing knowledge and competence in the early detection of EADs is fundamental for physicians, especially pediatricians and general practitioners, to ensure the creation of appropriate intervention networks to support patients and families and to initiate a multidisciplinary and evidence-based treatment [8]. The paper proposes the opinion of the pediatrician and child neuropsychiatrist regarding the identification and management of two conditions belonging to the EADs chapter: ARFIDs and atypical anorexia nervosa (AN). Both ARFIDs and atypical forms of anorexia can manifest early and insidiously; they can be underestimated, underdiagnosed and untreated, thus causing severe malnutrition over time, and can be complicated by the presence of psychiatric comorbidities, thus requiring a multidisciplinary management from early childhood [9,10].

## 2. Atypical Anorexia Nervosa

### 2.1. The Child Neuropsychiatrist’s Point of View

Eating disorders include a number of ‘atypical’ presentations. Specifically, we refer to atypical EADs when the symptomatology presents in an attenuated pattern. Affected patients present a low frequency of purging behavior or binge eating, the Body Mass Index (BMI) is in the normal range, thoughts about body shape and weight are denied or mainly symptoms of a somatic nature can be manifested [11]. Atypical forms of anorexia nervosa are newly introduced in the DSM-5. With this term, we refer to a disorder characterized by the presence of an altered body image without significant weight loss or with a weight that is still within the normal range for the age [12]. One of the main issues related to atypical AN is indeed the presence of a normal weight: this characteristic often leads to a delay in the care provided to patients. This is mainly related to the underestimation of these forms by clinicians despite the numerous medical complications that individuals are vulnerable to [13]. For example, a useful clinical clue in the diagnosis of atypical AN derives from the frequent presence of previous obesity or overweight. The subsequent initiation of a diet, linked to reinforcements derived both from a better perception of the own body image and from environmental stimuli (e.g., positive reinforcement from the family), can subsequently lead to the onset and maintenance of this disorder [14]. However, culturally, the idea that ‘anorexic thinking’ in itself constitutes a psychiatric problem still seems to be uncommon. Therefore, the assessment of BMI alone is likely to become the only guide for the clinician, who may thus fall into the misconceptions of both the psychiatric and medical condition. In addition, the possibility of developing comorbidities appears to be the same as in subjects with “typical” AN: specifically, the most prevalent comorbidities are depressive symptoms, obsessive-compulsive disorder and suicidal ideation/self-harm [15]. As with the classic forms of AN, family-based treatment is the first-line therapy in atypical forms [14]. It is also well known that the psychopharmacological approach in cases of AN has mixed results: mainly, however, it appears to have a minimal effect both in terms of improving anorexic symptoms and weight recovery [16]. There are currently no studies in the literature evaluating the possible effectiveness of psychopharmacological treatment in atypical forms.

### 2.2. The Pediatrician’s Point of View

Eating disorders in childhood are very common problems. In fact, it is estimated that around 25% of children with normal psychophysical development and 80% of children with developmental delay may present a nutrition and eating disorder [17]. It may manifest as an inability to feed adequately, resulting in difficulty gaining weight or significant growth retardation. The highest risk groups are preterm infants, those with a birth weight below the 10th percentile for gestational age, children with craniofacial abnormalities and/or genetic syndromes. There are often typical patterns of food refusal, characterized by oppositional attitudes on the part of the child (pushing away or throwing away food, crying when offered and then at the sight of the bottle) or their apparent lack of interest in food (falling asleep and stopping eating, holding food in their mouth). In general, an eating disorder may begin between 6 months and 4 years of age when self-feeding is attempted, but also at later stages of life when the imbalance between the subject’s real energy intake and needs is more evident. Atypical forms of AN exist and the medical complications of this disorder are not always and easily identified. A study of 118 individuals, 59 meeting DSM-IV criteria for AN and another 59 with ‘subthreshold’ AN, showed no difference in physical parameters on presentation, with the exception of the lower white blood cell count in the AN [18] group. It is also the authors’ experience that individuals with AN and atypical AN share similar degrees of caloric restriction and malnutrition, and thus have overlapping clinical pictures. 

## 3. The “Future” of Avoidant/Restrictive Food Intake Disorders

### 3.1. The Child Neuropsychiatrist’s Point of View

ARFIDs refer to clinical manifestations characterized by disturbance of nutrition or eating that occurs in the inability to meet appropriate nutritional/energy demands. A significant weight loss and/or nutritional deficit, need for supplementary feeding (oral or parenteral) and marked interference with psychosocial functioning are associated [2]. Specifically, three distinct subtypes of manifestations are described: avoidance of food based on sensory characteristics (shape, color, packaging and so on); avoidance secondary to the presence of phobic symptoms (e.g., after choking episodes); finally, avoidance may be related to ‘disinterest’ in food [19]. ARFIDs have an estimated prevalence between 5% and 14%; the most frequent presenting symptoms are: decreasing portion sizes, avoiding specific foods, history of nausea, early satiety [20]. Furthermore, it is necessary to pay attention to possible associated diagnoses: ARFIDs are very often accompanied by generalized anxiety disorders, obsessive-compulsive disorders [21] and, especially in manifestations of selectivity, autism spectrum disorders; in this case, the presence of ARFIDs can be one of the possible red flags in mild forms, which can be more difficult to detect early [22]. Although ARFIDs are typically associated with low weight, this is not a necessary condition, as nutritional deficits may also be associated with overweight or obesity: for example, a child who completely excludes plant foods from the diet may be more prone to overweight, but still experience significant nutritional deficiencies [23]. The impact of ARFIDs on individuals appears to be significant with repercussions not only nutritional but also related to quality of life and psychosocial functioning [24]; therefore, a multidisciplinary and integrated approach that can assess this disorder from different perspectives is required. The patient with ARFIDs must be initiated into a rehabilitation project that takes into account not only the subject but also the entire family: among the various techniques that have proved most beneficial are food chaining, family-based therapy and cognitive-behavioral therapy [19].With regard to psychopharmacological treatment, there is preliminary evidence in the literature regarding the use of selective serotonin reuptake inhibitors (SSRIs): to date, however, there is still little evidence to define an univocal pharmacological approach in the case of ARFIDs [25,26].

### 3.2. The Pediatrician’s Point of View

It is not uncommon for pediatricians to find children with a benign condition often referred to as ‘picky/fussy eater’ in which caloric input is preserved at an adequate weight and with spontaneous resolution during childhood and adolescence [27]. The peak at which this disorder occurs is between 2–6 years of age but it can affect 14–50% of school-age children and 7–27% of older children. Only in a subgroup of children, selective feeding can significantly impair growth and development and meet the diagnostic criteria of ARFID. When a child at risk of malnutrition or with a severe picture of overt malnutrition has been identified, nutritional intervention is an important strategy. This intervention can be carried out initially by increasing only the foods that the child prefers. However, oral nutritional supplements (ONS) can be added both to compensate for the caloric intake and to correct any selective macro- and micronutrient deficits. If these strategies are not sufficient, these children may need to be sent to specialized artificial nutrition centers where combined strategies of enteral and/or parenteral nutrition can provide the necessary time for the child neuropsychiatrist to act on the disorder and at the same time for the pediatrician to exclude irreparable risks to the child’s nutritional status.

## 4. Questions to the Expert

### 4.1. The Pediatrician Replies

1. *What are the most common medical conditions to be considered in a differential diagnosis of weight loss in adolescence?*

In most cases nutritional disorders are transient, but in 3–10% they may be associated with a risk of malnutrition. In the differential diagnosis, certain organic causes such as food allergies, gastroesophageal reflux disease, swallowing disorders and malabsorption causes (celiac disease, cystic fibrosis, chronic inflammatory bowel disease, etc.) must always be taken into account. The clinical approach must be reassuring and ‘corrective’ in most cases, although any warning signs (vomiting, psychomotor retardation, etc.) or a tendency towards poor growth or malnutrition must be highlighted. Screening for poor growth/low weight and feeding difficulties should be part of regular pediatric check-ups.

2. *What are the medical complications to be considered as most dangerous in cases of malnutrition and how to manage them?*

For the child, growth impairment, first ponderal and later statural, represents serious, often irreparable damage. Pubertal retardation may be difficult to recover from. Selective deficiencies and severe dehydration can seriously impair growth and health.

3. *The increase in eating disorders, especially in their more subtle forms, has also seen an increase in the recurrence of diseases ‘of the past’, which are now uncommon and therefore more difficult to recognize and treat. What could be the ‘rare’ conditions related to malnutrition, how should they be hypothesized and how should they be managed?*

Often, malnutrition pictures are so selective and severe that nutritional deficits that have disappeared, such as Beriberi or Scurvy, re-emerge. It is of fundamental importance in the pediatric objective examination to pay attention to almost forgotten clinical signs (gingival hypertrophy, bleeding gums, anemia, hypochromia, walking difficulties, mental confusion) and correlate them with nutritional deficits by paying attention to the patient’s food diary, which generally, if well done, testifies to food selectivity and suggests possible related deficits.

The assessment of the nutritional and biochemical status and the dosage of vitamins is the next step for a correct diagnosis and the initiation of a timely restoration of the deficit.

### 4.2. The Child Neuropsychiatrist Replies

1. *What can be the easiest and most effective way for the pediatrician to detect possible eating problems in the absence of clear signs of malnutrition.*

One of the main prejudices in the diagnosis of an eating disorder is that doctors do not take an adequate dietary history. If they do not ask specific questions about nutrition, in most cases the problems go unreported. Especially with adolescents, also considering the lowering of the age of onset of eating disorders, performing more accurate interviews about eating can become crucial in identifying the onset of diseases or conditions that require attention.

2. *What physical conditions are risk factors for the development of an eating disorder in adolescence?*

Nutrition and eating disorders are characterized by the presence of a persistent dysfunction in the consumption or absorption of food. Such conditions affect the physical health and the psychological and social functioning of the affected individual. Therefore, all clinical conditions that imply the adoption of particular diets, such as diseases with a metabolic-genetic basis (juvenile diabetes, phenylketonuria, Wilson’s disease, etc.) or chronic gastrointestinal pathologies (Chron’s disease, ulcerative rectocolitis, short bowel syndromes, etc.), may in themselves represent ‘predisposing’ conditions for the development of an eating disorder. For this reason, these conditions deserve special attention from the treating pediatrician; they also require periodic follow-up of the psychological aspects linked to the disease presented, for the possible early detection of psychiatric symptoms to be referred to the child neuropsychiatrist. Last, but not least, all those clinical conditions in which the approach to food, starting from the early stages of weaning, may be difficult or ‘atypical’ are to be monitored: children who experience early access to artificial nutrition, may manifest over time not only problems related to oral-occlusal-facial praxis and chewing, with consequent difficulties in the intake of solid foods, but also difficulties in developing ‘pleasure’ for food. Such conditions are at risk of progressively developing restrictive or avoidant ways of eating.

3. *What indications can be offered to parents of children and adolescents who present difficulties in their approach to food or eating problems?*

It is important, as all potentially debilitating high-risk diseases, to work from a preventive point of view, i.e., to educate parents and children, from an early age, in healthy and correct nutrition. However, a parent’s anxiety about a child who does not eat can often lead to the triggering of dysfunctional dynamics at mealtimes and the adoption of coping behaviors that risk becoming disease-causing themselves. In general, restoring the parent’s educational role in the eating becomes fundamental. The meal, often experienced as a moment of great stress, must be transformed into a moment of sharing and conviviality. It is good to invite all members of the family to eat together and all the same dishes, without differentiating where possible. It is good that the meal is eaten in a reasonable time and does not go on for too long (keeping the plate at the table for an unlimited time waiting for the child to finish the portion is not useful). Invite, therefore, the child to try new and unwanted foods, so that they can always experiment with their own taste, without satisfying the demands of a few habitual foods. Also invite them to have five meals a day, making sure that the snacks (mid-morning and mid-afternoon) are small and consist of healthy foods, to avoid the sense of hunger being satisfied with “non-foods”, arriving at the main mealtime with little motivation.

## 5. Concluding Remarks

Children with EADs should be identified early given the increasing number of affected individuals in pediatric age. Identifying possible risk factors or behaviors that signal the possibility that a EADs is arising is essential (see Table 1).

In developmental age, atypical eating behaviors can be managed by nutrition and eating education interventions, starting from the pediatrician’s outpatient clinic. Nevertheless, when there are alarms for the presence of an EADs, the multidisciplinary approach is necessary; therefore, patients should be referred to specialist centers where they can be followed by different professionals (pediatrician/gastroenterologist and/or nutritionist specialist, child neuropsychiatrist, speech therapist, rehabilitation therapists). The objectives of rehabilitation intervention must aim not only at nutritional aspects (ensuring adequate calories, protein and other nutrients, identification of forgotten nutritional deficits), but above all at a correct and timely psychiatric diagnosis (see Table 2). The integrated approach between the various specialists guarantees better outcomes for the patient and allows a targeted intervention to the issues [28].

## Author Contributions

Conceptualization, M.I.S. and M.P.R.; methodology, A.S.; validation, M.I.S., C.B. and M.P.R.; formal analysis, R.G.; investigation, M.P.R.; resources, A.S.; data curation, R.G.; writing—original draft preparation, M.P.R.; writing—review and editing, M.I.S.; supervision, M.I.S.; funding acquisition, C.B. All authors have read and agreed to the published version of the manuscript.

## Figures and Tables

**Table 1:** Red flags for eating disorders.

The child eats only his favorite foods
Most of the calories assumed are from liquid
The child is distracted while eating
Child eats food hidden in other foods or liquids
Meals last more than 30 min
Excessive concern for one’s weight and body shape
Restriction in food intake (reduction in total intake or consumption of certain foods, with particular avoidance/fear of fatty foods) or verbalizations concerning the fear of gaining weight
Compulsive physical exercise (with increasing anxiety related to the eventual impossibility of completing what is predetermined/established)
Guilt at mealtimes or shame on eating in the presence of others

**Table 2:** Role of the pediatrician and child neuropsychiatrist in ARFID and atypical anorexia.

Denomination	Main Clinical Features	Age and Prevalent Conditions of Presentation	Role of the Pediatrician in Diagnosis	Role of the Child neuropsychiatrist in Diagnosis	Role of the Pediatrician in Treatment	Role of the Child neuropsychiatrist in the Treatment
**Avoidant/restrictive food intake disorder**	Food restriction from apparent lack of interest, avoidance from sensory characteristics or for the presence of phobic aspects that requires artificial nutrition support	Mainly early and second childhood (school age), with risk of chronicization in adolescence and later in adulthood	Early detection Exclusion of organic causes Referral to the child neuropsychiatrist specialist Referral to a clinical nutrition center, specialized where appropriate	Diagnostic confirmation on suspected dispatch Evaluation of other psychiatric comorbidities/neurodevelopmental conditions	Monitoring of growth and/or dietary trend/nutritional status	Treatment indications Management and psychopharmacological of comorbidities if present and follow-up if appropriate
**Nutrition or eating disorder** **with other specification**	Included in this category: atypical anorexia nervosa; bulimia nervosa of low frequency and/or limited duration; binge-eating disorder with low frequency and/or limited duration; elimination conduct disorder; nocturnal feeding syndrome	Mainly adolescence, but current lowering of age of onset, especially in comorbid cases with other psychiatric/organic conditions and with subtle and deceptive manifestation	Early detection Exclusion of medical causes of malnutritionReferral to the child neuropsychiatrist specialist Referral to a clinical nutrition center, specialized where appropriate	Diagnostic confirmation on suspected sending Evaluation of other psychiatric comorbidities	Monitoring of dietary trends/nutritional status	Multidisciplinary management in specialized facilities or reference centers Treatment indications Psychopharmacological management of the disorder and comorbidities if present and follow-up if appropriate

## Data Availability

Not applicable.
